# Harnessing nature and ancient wisdom to store carbon

**DOI:** 10.1073/pnas.2305214120

**Published:** 2023-05-01

**Authors:** Hugh Helferty

**Affiliations:** ^a^Queen's University, Kingston, ON K7L 3N6, Canada

There is broad agreement that reversing the rise in CO_2_ concentration in the atmosphere is critical to avoiding a climate disaster. To accomplish this, robust carbon-negative solutions are needed in addition to technologies that eliminate or reduce CO_2_ emissions. These solutions fall into two groups: “engineered” approaches, such as direct air capture (DAC), and nature-based solutions. While DAC suffers from high cost, it has the advantage of enabling permanent CO_2_ sequestration deep underground. Most nature-based solutions, such as growing trees, are cheaper but provide only temporary storage (tens or at most several hundred years) before the carbon is re-released to the atmosphere. In this issue of PNAS (1), two Berkeley researchers, Yablonovitch and Deckman, propose an inexpensive, scalable approach to transforming nature-based solutions from temporary to permanent storage.

The proposed technology, called Agro-Sequestration, grows biomass crops (plants and trees) to capture carbon from the air, then buries harvested vegetation that has been dried and salted in engineered biolandfills. This Agro-Sequestration proposal creates an invaluable new option for tackling climate change that could be scaled to remove and sequester the equivalent of 20 Gt of CO_2_/y.

The article points out that there have been several proposals to bury biomass in wet environments where some degree of anaerobic decomposition into CO_2_ and CH_4_ greenhouse gasses would be expected. The first of these was from the physicist Freeman Dyson who pointed out (2) that the productivity of plants and trees is sufficient to offset the world’s CO_2_ emissions. Biofuel research has since developed energy crops that are significantly more productive than those considered by Dyson.

To eliminate sequestered biomass decay, the authors took inspiration from a food preservation technique dating back to Babylonian times, namely dryness and salting. They show that dryness assisted by salt is expected to extinguish all life when the moisture level in the sequestered biomass is low enough. Moisture levels are measured by a quantity similar to relative humidity called “water activity.” Quantitatively, they predict that if water activity is consistently below 60%, all life comes to an end in aerobic as well as anoxic and anaerobic environments. This is the concept underpinning the Agro-Sequestration approach which is meant to preserve ~100% of sequestered biomass for thousands of years.

From the archeological record Yablonovitch and Deckman point to a date palm named Methuselah as proof that biomass, if kept sufficiently dry, can be preserved well beyond the next millennium. In the 1960s, Israeli archaeologist Yigal Yadin (3) discovered date palm seeds among ancient ruins atop Masada, a mesa overlooking the Dead Sea—one of the most arid places in the world. Since 1965, the seeds remained in a drawer until a doctor researching natural medicines, Sarah Sallon, requested them in 2005. She then had the seeds carbon-dated and learned that they were indeed 2,000 y old (4). Then, she asked horticulturist Elaine Solowey to plant them. They germinated (5), and Methuselah, one of those date palm trees, continues to thrive today. Although this and other archeological records are impressive, there is a need for more research into the exact level of dryness that is needed to suppress biomass degradation. Is a water activity of 0.75 (which can be obtained with NaCl salt) sufficient? This is but one of many questions that can be researched, but the evidence presented is sufficient to merit construction of a series of well-instrumented demonstration biolandfills. An appealing feature of this approach is that with their biolandfill designs, instrumentation would be able to confirm the ability to suppress sequestered biomass degradation in real-world settings. This appears to be a compelling low-risk approach that would facilitate rapid deployment while other research proceeds.

An attractive feature of the Agro-Sequestration highlighted in the paper is its relative simplicity and overall efficiency. A metric proposed to quantify efficiency that appears to be particularly useful for carbon negative processes is 

[1] $$\mathrm{Carbon}\;\mathrm{Efficiency}\;\left(t\right)=\frac{C_{\mathrm{available}}{-C}_{\mathrm{lost}}\left(t\right)}{C_{\mathrm{available}}},$$

where C_available_ is the total amount of carbon initially available to the capture process, and $C_{\mathrm{lost}}\left(t\right)$ is the net carbon lost in the capture and sequestration process. $C_{\mathrm{lost}}\left(t\right)$ includes carbon that was available but not sequestered, direct and indirect emissions during the capture and sequestration process, credits for carbon sequestered in a different manner, and the time-dependent total fugitive emissions from degradation of the sequestered carbon. For most technologies, there are no or minimal credits making the net carbon loss ( $C_{\mathrm{lost}}$) negative and concomitantly the carbon efficiency less than one. Agro-sequestration is shown to have an initial carbon efficiency in the range 0.9 to 1.05 of the carbon available in the grown biomass. The value of 1.05 is associated with potential credits for carbon sequestered in soil and plant roots that is not accounted for in the harvested biomass. Without including this credit, the carbon efficiency is constant with time because the sequestration is expected to be stable for thousands of years. Long-term stability is due to a water diffusion barrier that encases the sequestered salted dry biomass. This diffusion barrier contains two nested 2-mm-thick polyethylene geomembranes that limit ground water ingress to less than the equivalent of a yearly addition of a ~1.75-μm-thick water layer.

The authors applied this metric to biofuels and concluded that Agro-Sequestration would be more carbon efficient than biofuel production with carbon capture (BECCS). A consequence of this is that less land area would be needed for CO_2_ drawdown by Agro-Sequestration compared to BECCS. This reduction would lessen social burdens that have been discussed for scenarios where BECCS has been proposed to offset a significant fraction of world emissions. They predict that land area requirements will decrease even more in the future because of a Moore’s law behavior for agricultural productivity. In particular, the Moore’s law behavior they show has crop yields (ton/hectare) doubling every 50 y, reducing land area requirement and likely agricultural costs in the future.

The proposed technology, called Agro-Sequestration, grows biomass crops (plants and trees) to capture carbon from the air, then buries harvested vegetation that has been dried and salted in engineered biolandfills.

Carbon efficiency could be more widely used to provide an additional perspective on other carbon-negative capture and sequestration technologies because it accounts for degradation of sequestered carbon over time. Integrated with a climate model, it could add additional perspective on the relative climate impact of technologies that capture and stably sequester carbon compared to those that provide a near-term mitigation but emit greenhouse gasses in the long term. Several valid questions arise from applying this metric to less-stable nature-based solutions discussed in the paper. For example, how rapidly does the carbon efficiency of reforestation and afforestation drop from ~1 toward 0 from the death of trees that would begin 10 to 80 y after planting? Carbon stored in soil and roots from carbon farming is an intriguing technology that might have a carbon efficiency in a range from ~0.01 to ~0.1 of the total carbon in the grown crop, but how much and how rapidly would it drop with time due to degradation from microbes, insects, or fungi? These are but a few of the questions that can be selected from the wide suite of nature-based solutions being deployed. For all nature-based solutions, there is an acute need for research to more accurately quantify their carbon efficiency along with their climate impact on longer time horizons (for example, 10, 20, 50, and >100 y after they have been implemented).

Nature-based solutions tend to be significantly less expensive than technologies that capture carbon in the form of CO_2_ directly from the atmosphere or from industrial emissions and sequester it either in underground geological formations or as a mineral. Verification and study of the stability of these sequestrations continue, but broadly, it is thought that they will be stable and hence the carbon efficiency for them will be constant with time. Because these technologies are still in early stages of deployment with significant efforts to increase their use, it remains extremely important to continue studying and verifying the ability to stably trap CO_2_ in underground formations or mineralize it. In addition, it is vitally important to gain a better perspective on the relative values of climate mitigation tools that provide a stable sequestration and those that would in the future emit greenhouse gasses from sequestered carbon.

An even more important metric than carbon efficiency is the total cost of carbon capture and sequestration. For Agro-Sequestration, this is calculated to be ~US$60/ton of CO_2_ from a bottom-up cost analysis as well as from a scaling argument based on current agriculture and landfill costs. Numerically, this translates to a premium of US$0.53/gallon of gasoline. At this price, offsetting 20 Gt of the world’s carbon dioxide emissions would set back the world economy by 2.4%. This price for capture and sequestration appears to be at the lower end of the range of costs for technologies being explored to capture and sequester CO_2_ from industrial emissions, directly from the air, or with BECCS. It would significantly reduce projections of CO_2_ capture and sequestration costs, which in many scenarios are expected to be US$300–1,000/ton by 2050 (6). Concomitantly, the low projected cost and potential for rapid scaling would dramatically increase the likelihood of achieving “net-zero” by 2050.

To some extent, markets for CO_2_ credits could advance the new Agro-Sequestration option, but a mandate on producers to sequester the CO_2_ they produce would minimize the use of credits as an excuse for continued emissions. There are several possible approaches that already exist, such as carbon capture and storage, carbon farming, planting trees, or sequestering carbon in products like plastic or cement. Most likely, more significant advancements will require robust regulatory mandates that support carbon negativity. For example, a framework with a form of carbon tax could enhance the viability of “Carbon Takeback” (6) where producers take responsibility and reverse all emissions that result from their products and activities.

As with all technologies, the most important next steps for Agro-Sequestration are demonstration and further validation of the claimed advantages. Construction of biolandfills sequestering a variety of biomass sources in different locations would provide initial steps on an experience curve and lead to improvements and cost reductions. Such early demonstration projects would have to be instrumented to provide data to establish efficacy of this approach for governments, regulators, and markets.
